# The Gantry as a Drive for a Horizontal Bike: Initial Investigation of Rotary Work

**DOI:** 10.1155/2021/6654377

**Published:** 2021-01-23

**Authors:** Łukasz Bereś, Paweł Pyrzanowski

**Affiliations:** ^1^Warsaw University of Technology, Institute of Aeronautics and Applied Mechanics, Nowowiejska str. 24, 00-665 Warsaw, Poland; ^2^BereSolutions Łukasz Bereś, https://www.beresolutions.com/, Warsaw, Poland

## Abstract

The gantry drive (also, “the gantry”) is a mechanism that receives human-generated mechanical energy. The gantry used in a horizontal bike is a type of drive, and it is an alternative to a typical crankset. The purpose of this paper was to compare rotary work generated by the gantry and the crankset. The comparative criterion for the gantry and the crankset was work in rotational motion. The comparison was based on static tests; forces put into both drive systems were measured, and the rotary work was mathematically calculated. The forces put into the drive systems were measured for a man 177 cm tall and of 76 kg mass. To facilitate analysis and tests, the first gear wheel to receive force from the toothed rack (the gantry drive) was assumed to have the same radius as the crank (the crankset drive). Mathematical analysis performed for one full rotation (360°) of the first gear wheel and crankset showed that rotary work for the gantry was 2117.31 J and for the crankset 804.81 J. Ultimately, it was shown that the gantry can better receive mechanical energy from the human than the crankset. This means that a human will be less tired when riding a horizontal bike equipped with the gantry compared to a horizontal bike equipped with the crankset; assuming that in both cases, the bike speed is the same. Additionally, thanks to the use of the gantry drive in a horizontal bike, it is possible to achieve higher speeds compared to a horizontal bike equipped with the crankset.

## 1. Introduction

The gantry drive is a mechanism in which the linear movement of the toothed rack is converted into the rotational movement of the first gear wheel. The linear movement of the gantry is carried out with linear bearings that move along fixed guides, whereby the toothed rack is permanently connected to the gantry. Construction details are presented below.

**Figure 1:**
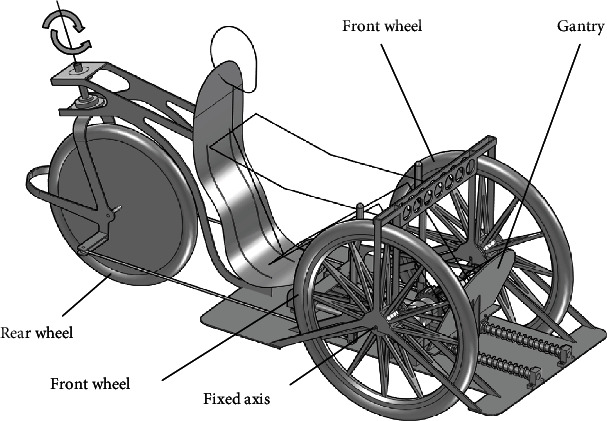
The gantry used in the horizontal bike.

**Figure 2:**
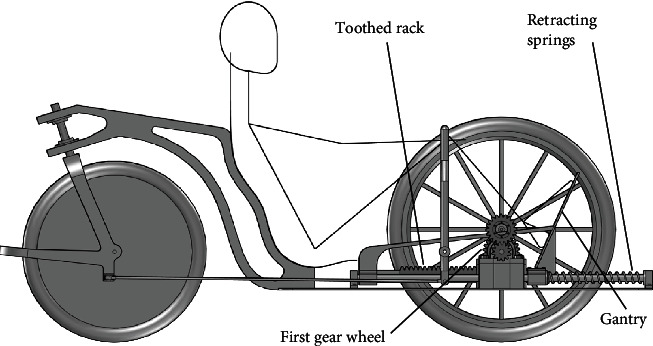
The gantry used in the horizontal bike (side view).

**Figure 3:**
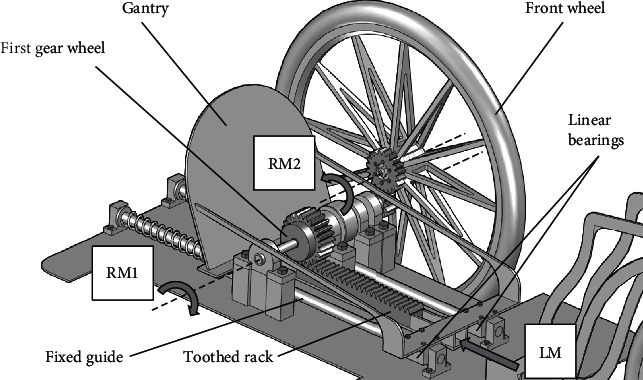
The gantry drive in the horizontal bike (general view).

**Figure 4:**
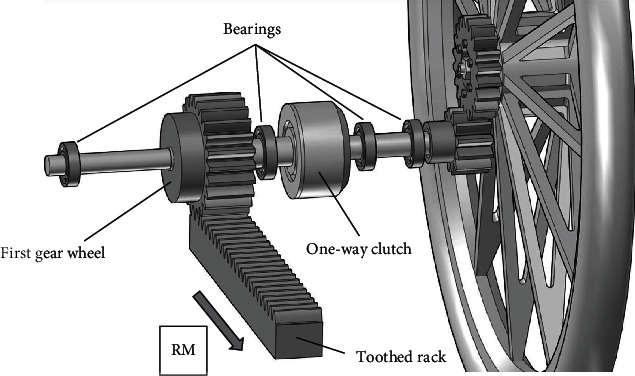
The gear transmission in the horizontal bike (detailed view).

The gantry used in a horizontal bike is a type of drive, and it is an alternative to a typical crankset. The design of such a horizontal bike equipped with the gantry is shown in Figures 1 and 2. The detailed design of the gantry drive is shown in Figures 3 and 4.

The suspension of the horizontal bike presented in Figure 1 was designed in such a way that the front wheels are fixed axis and the rear wheel is used to turn the bike. Thanks to these features and transmission of drive to only one of the front wheels, the drive system does not require articulated joints and bellows couplings. The drive system is rigid and has high efficiency.

Figures 1 and 2 show an example of using the gantry as a drive in a 3-wheeled bike. Nevertheless, the gantry can be used as a drive for other vehicles powered by human muscles (e.g., 4-wheel bikes and boats).

A man presses his feet on the gantry (transfer of force), thus causing linear movement (LM) of the toothed rack. Movement of the toothed rack causes rotation of the first gear wheel (RM1). Then, the drive is transmitted to the bike wheel (RM2) through a gear transmission. The gear transmission shown in Figures 3 and 4 is an example of how to transfer the drive from the first gear wheel to the bike wheel. There are many combinations of how it is possible. By adding more gears, it is possible to achieve additional gear ratios and obtain reverse gear in the bike.

The gantry can be a drive for a horizontal bike because a one-way clutch is used in the drive system. Thanks to the one-way clutch between the first gear wheel and the bike wheel, retracting movement (RM) of the toothed rack is possible when the horizontal bike moves forward. When the toothed rack begins to move backwards, the one-way clutch disengages the drive system and the toothed rack can be freely retracted.

The gantry and the crankset can be tested and compared in two ways:

- *Static Approach*. Forces put into the drive system are measured and the rotary work is mathematically calculated
- *Dynamic Approach*. The driving torque is measured and the energy expenditure of the man is measured

In the work described here, only static tests were performed.

The solution of using the gantry as a drive, especially in 3- and 4-wheeled bikes, was presented in the patent application [1]. Moreover, two other patent applications related to the gantry have been filed. The first of the additional patent applications [2] describes how the drive from the gantry is transferred to the bike wheel, and the second one [3] describes a solution that supports the operation of the gantry by using elastic elements that retract the gantry after the work phase.

## 2. Materials and Methods

The leg-driven crankset is a widely used mechanism in horizontal bikes and is widely known, which is why it has been compared with the gantry. This comparison allows somehow to classify the gantry used as a drive mechanism.

The rotary work was used to compare the gantry with the crankset. Calculation of work in rotational motion requires only knowledge of the forces generated by man in subsequent phases of movement of mechanisms. Force measurement (static approach) is relatively easy to carry out, which is why this approach was used in the initial gantry analysis.

The gantry was developed on the basis of observations that took place in a gym. It has been observed that the person exercising on a gantry is able to lift a very heavyweight. Greater force means that more energy can be accumulated assuming the same displacement for the acting force (general formula for energy—see equation (1)). A bike needs a drive torque to move. In turn, the torque arises as a result of force acting on a radius. To somehow compare the typical crankset with the gantry in further analysis, the assumption was made that the radius for introducing force into the crankset and the gantry will be the same—which means that the length of both cranks is identical as the pitch diameter of the first gear. In addition, assuming for analysis that the first gear wheel that receives force from the toothed rack (crankset drive) has the same radius as the gantry (gantry drive) greatly simplifies the calculations.

General formula for energy is the following [4–6]:

(1) $$\begin{array}{c} E=F\;s, \end{array}$$

where *E* [*J*] is the energy, *F* [*N*] is the force, and *s* [*m*] is the displacement.

**Figure 5:**
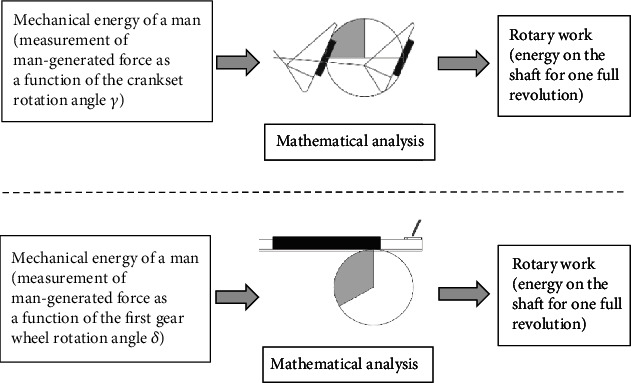
Schematic workflow for the crankset drive (top) and the gantry drive (bottom).

Figure 5 schematically shows the tests and analysis that have been carried out for the crankset and the gantry. The tests were aimed at measuring what forces a human being can generate as a function of the angle of rotation of mechanisms. During the test, the angles between the torso and the thigh, as well as the thigh and the calf were measured—details are described below in this chapter [7]. Based on the test data, analyses were carried out, the final result of which is determination of work in rotational motion. Rotary work in calculations is energy that can be received for one complete rotation of the shaft (the crankset or the first gear wheel of the gantry).

**Figure 6:**
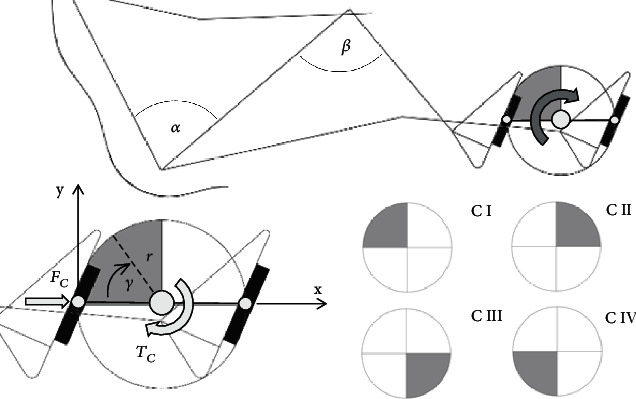
The crankset—model for analysis.

**Figure 7:**
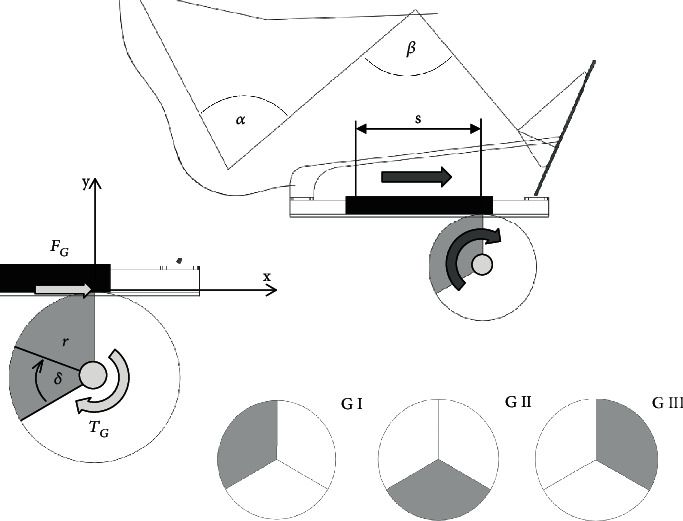
The gantry—model for analysis.

All mathematical analyses were made on the basis of theoretical models of mechanisms presented in Figures 6 and 7.

Figure 6 shows the model for the crankset analysis. When the crankset is moving, the *α* and *β* angles change. The *α* angle is the angle between the torso and the thigh. In turn, the *β* angle is the angle between the thigh and the calf. Force *F*_*C*_ is the force that is introduced into the system. The point of application of force *F*_*C*_ changes as the *γ* angle changes. The *γ* angle changes in the range from 0° to 360°, and with the change of this angle, the crankset goes into the subsequent phases of the movement C I, C II, C III, and C IV.

Figure 7 shows the model for the gantry analysis. When the gantry is moving, the *α* and *β* angles change. The *α* angle is the angle between the torso and the thigh. In turn, the *β* angle is the angle between the thigh and the calf. Force *F*_*G*_ is the force that is introduced into the system. The point of application of force *F*_*G*_ does not change with the change of angle *δ*. The *δ* angle changes in the range from 0° to 360°, and with the change of this angle, the first gear wheel goes into the subsequent phases of the movement G I, G II, and G III.

In order to simplify the analysis, a number of assumptions have been made, and some simplifications have been introduced, as detailed below:

- The rotary work was compared to one full turn of the crankset and of the first gear wheel, i.e., *γ* = *δ* = 360° = 2 *π* rad
- The radius of the crank and the first gear wheel is equal to *r* = 0.17 m. The radius 0.17 m is the typical crank length in bikes. Theoretically, the radius can be increased indefinitely, which would have a very beneficial effect for the torque. Nevertheless, due to the structure of the human skeleton, this is not possible; as shown in the next chapter, in the extreme positions of the mechanisms, the measured force is observed to decrease
- The stroke of the toothed rack was chosen so that it is equal to 1/3 of the circumference for the pitch radius in the first gear wheel. The stroke was chosen with the trial and error method. It is known that the crank is 0.17 m, so the diameter of the crankset is 0.34 m and it was in this area that the stroke was sought; the aim was to maintain similarity of the extreme positions of human limbs. The stroke found was a little larger than the diameter of the crankset; the pitch is 0.36 m

(2) $$\begin{array}{c} s=\frac{\text{Circuity}}{3}=\frac{2\;\pi \;r}{3}=\frac{2*\pi *0.17}{3}≅0.36\;\left[\text{m}\right]. \end{array}$$

(iv) The initial values of the *α* and *β* angles (the beginning of a given phase of the mechanism's operation) and the final values (the end of a given phase of the mechanism's operation) were selected so that the measured forces for the beginning and the end of a given phase of the mechanism's operation were similar. This approach was adopted based on preliminary measurements which are not described here. This approach seems right because it maximizes the energy stored on the output shafts

Simplifications for analysis that are not significant in the static approach or have little effect on the results:

- Change in potential energy of legs was omitted
- Bearing resistance and friction in mechanisms have been omitted
- Energy loss on the one-way clutch during retracting of the toothed rack has been omitted
- Downtime resulting from the need to retract the gantry has been omitted (energy needed to retract the gantry). Retracting the gantry is associated with overcoming the bearing resistance and the resistance generated by the one-way clutch. The prototype plans to use springs that will accumulate energy when extruding the gantry. Then, these springs will automatically withdraw the gantry. Construction details of the retracting springs are shown in Figure 2

**Figure 8:**
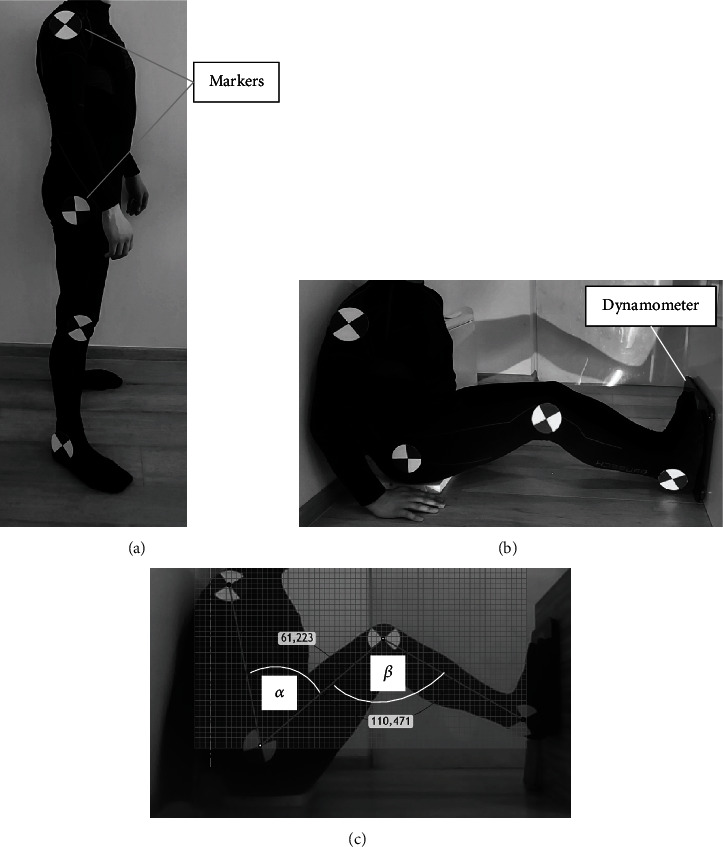
Pictures from tests: (a) position of markers on a man, (b) illustrative photo of the test and the position of the dynamometer, and (c) position C7 (see Table 1) when measuring force.

A dynamometer was used to measure the force during the test (see Figure 8(b)). The maximum force that a human was able to generate was measured. Mathematical analyses were performed for the maximum force. The measurement was made without shoes. During the force measurement, the position of the torso relative to the thigh and the position of the thigh relative to the calf were measured (*α* angle and *β* angle). Visual measurement techniques were used to measure *α* and *β* angles [7–9]. The markers were permanently attached to the clothes which the man was wearing. The markers were attached in such places that no side view was observed in translation (when moving the adjacent member, e.g., the thigh is immobilized and the calf moves); the markers were approximately attached at the pivot points of human skeleton bones. Angles (*α* and *β*) were determined using the CAD software (see Figure 8(c)).

Axes *x* and *y* are shown in Figures 6 and 7. Measuring coordinates *x* and *y* were determined on the basis of the construction of the crankset and the gantry. The calculation of measurement coordinates is presented below. Coordinates *x* and *y* were determined as a function of the *γ* angle (the crankset) and the *δ* angle (the gantry).

Measuring coordinates (*x*_*c*_, *y*_*c*_) for the crankset is as follows:

(3) $$\begin{array}{c} x_{c}=r-r\cos\gamma ,\\ y_{c}=\left|r\sin\gamma \right|. \end{array}$$

Measuring coordinates (*x*_*g*_, *y*_*g*_) for the gantry is as follows:

(4) $$\begin{array}{c} x_{g}=\frac{s}{8}*i, \end{array}$$

where *i* [1] is a number that varies from 0 to 8 for one phase (G I or G II or G III)

(5) $$\begin{array}{c} y_{g}=0. \end{array}$$

**Figure 9:**
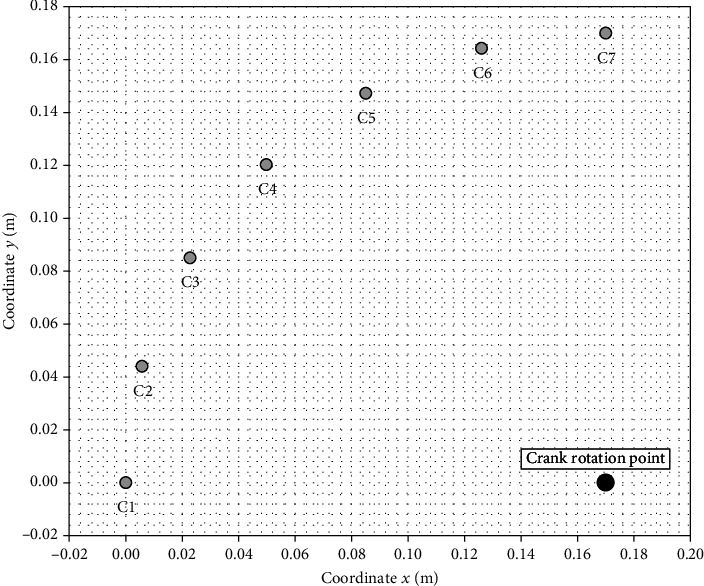
The measuring points for the crankset (phases C I and C III).

**Figure 10:**
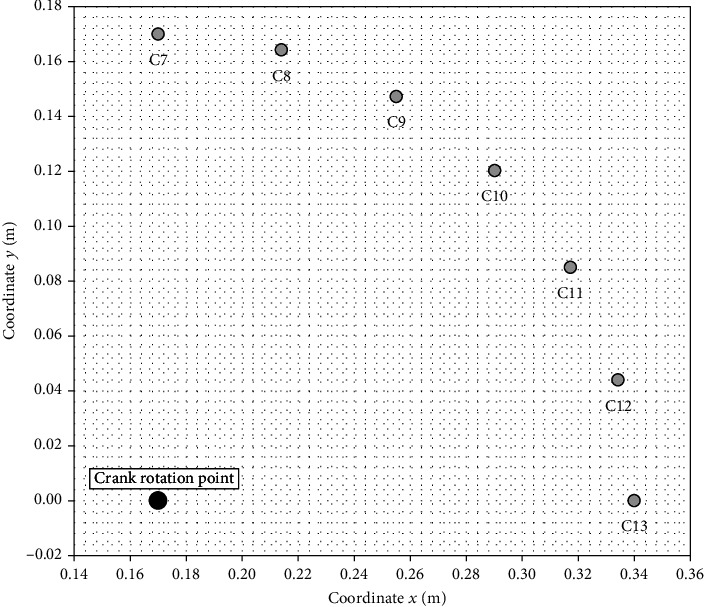
The measuring points for the crankset (phases C II and C IV).

**Figure 11:**
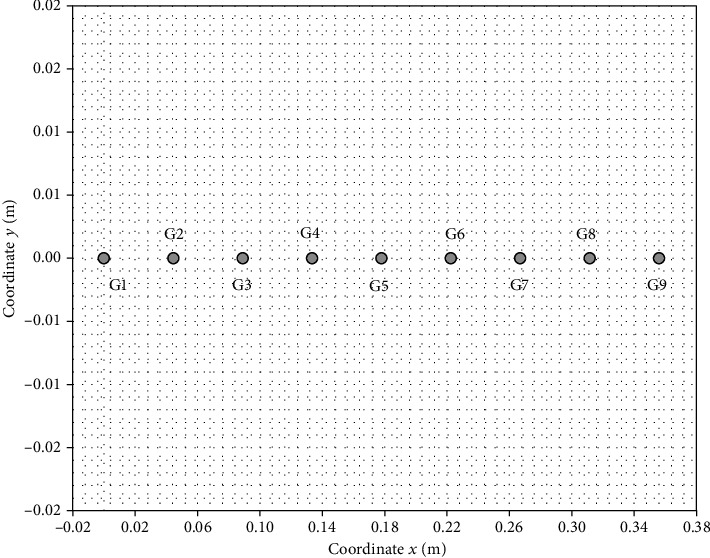
The measuring points for the gantry (phases G I, G II, and GIII are the same).

The calculations results are presented in tables in the next chapter. Figures 9 and 10 show the measuring points for the crankset. The point with the coordinates *x*_*c*_ = 0.17 m and *y*_*c*_ = 0 m in this figure shows the crank rotation point (see the large black dot in Figures 9 and 10).  Figure 11 shows the measuring points for the gantry.

Below are the formulas describing the torque generated by the crankset and the gantry as a function of the angle of rotation.

The torque generated by the crankset is as follows [4–6]:

(6) $$\begin{array}{c} T_{C}=F_{C}\;r_{c}, \end{array}$$

where *F*_*C*_ [*N*] is the force measured in tests for the crankset and *r*_*c*_ [*m*] is the active radius for the crankset.

Active radius for the crankset is the following:

(7) $$\begin{array}{c} r_{c}=y_{c}. \end{array}$$

The torque generated by the gantry is as follows [4–6]:

(8) $$\begin{array}{c} T_{G}=F_{G}\;r_{g}, \end{array}$$

where *F*_*G*_ [*N*] is the force measured in tests for the gantry and *r*_*g*_ [*m*] is the active radius for the gantry.

Active radius for the gantry:

(9) $$\begin{array}{c} r_{c}=0.17\;\left[m\right]. \end{array}$$

Below are the formulas describing the rotary work generated by the crankset and the gantry as a function of the angle of rotation.

The rotary work for the crankset is the following [10]:

(10) $$\begin{array}{c} W_{C}=\int_{0}^{2\pi }T_{C}\left(\gamma \right)\;d\gamma . \end{array}$$

The rotary work for the gantry is the following [10]:

(11) $$\begin{array}{c} W_{G}=\int_{0}^{2\pi }T_{G}\left(\delta \right)\;d\delta . \end{array}$$

All calculation results described in this chapter and test data are presented in Tables 1 and 2.

## 3. Results and Discussion

### 3.1. Results

The forces put into the drive systems were measured for a man 177 cm tall and of 76 kg mass. The crankset measurements were made (*F*_*C*_ force measurement) for phases C I and C II with the left leg, and for phases C III and C IV with the right leg—so as to simulate the operation of a real crankset. When measuring force *F*_*G*_, the force was generated by both legs simultaneously.

Table 1 summarizes all data from the analysis and test data for the crankset.  Table 2 summarizes all data from the analysis and test data for the gantry.

**Figure 12:**
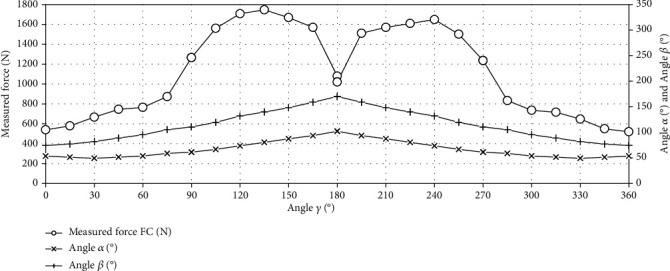
The measured force as a function of the angle *γ* (the crankset drive).

**Figure 13:**
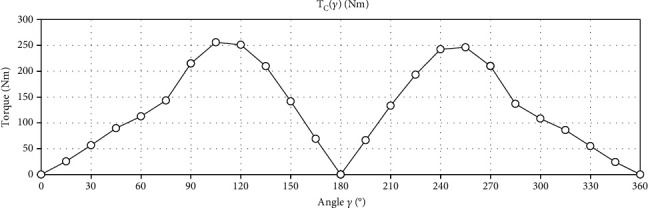
The torque as a function of the angle *γ* (the crankset drive).

The charts presented in Figures 12 and 13 were made on the basis of the data from Table 1. These graphs present data for the crankset drive.  Figure 12 shows the measured force as a function of angle *γ*.  Figure 13 shows the torque as a function of angle *γ*.

**Figure 14:**
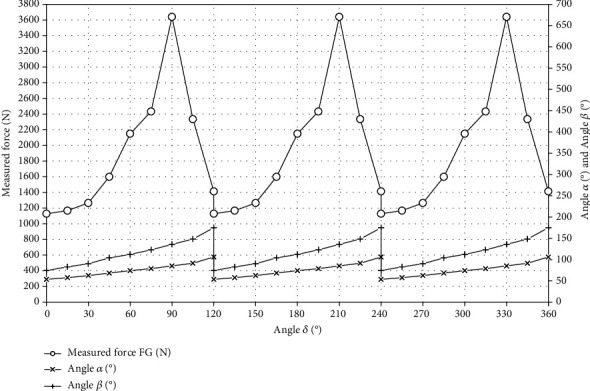
The measured force as a function of the angle *δ* (the gantry drive).

**Figure 15:**
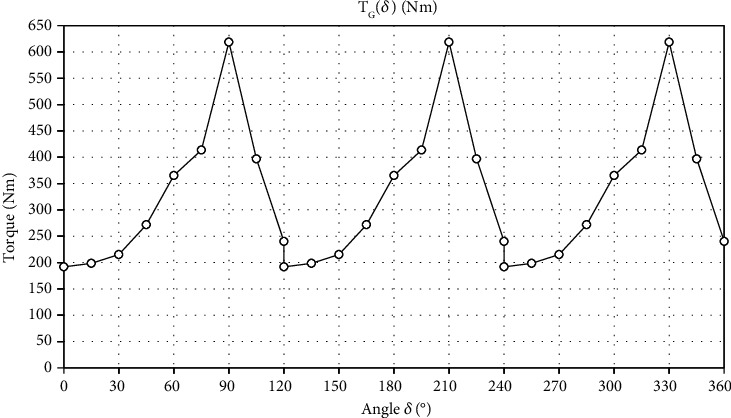
The torque as a function of the angle *δ* (the gantry drive).

The charts presented in Figures 14 and 15 were made on the basis of the data from Table 2. These graphs present data for the gantry drive.  Figure 14 shows the measured force as a function of angle *δ*.  Figure 15 shows the torque as a function of angle *δ*.

Figure 13 shows the torque as a function of angle *γ* (the crankset drive). The area under the curve is the rotary work for the crankset.

Figure 15 shows the torque as a function of angle *δ* (the gantry drive). The area under the curve is the rotary work for the gantry.

General formulas for counting the work in rotational motion performed for one full turn of the crankset and the first gear wheel are shown in the previous chapter (see equations (10) and (11)). The area under these curves was calculated as the sum of trapezoid fields.

The rotary work for the crankset is the following:

(12) $$\begin{array}{c} W_{C}=\int_{0}^{2\pi }T_{C}\left(\gamma \right)d\gamma =804.81\;\left[J\right]. \end{array}$$

The rotary work for the gantry is the following:

(13) $$\begin{array}{c} W_{G}=\int_{0}^{2\pi }T_{G}\left(\delta \right)d\delta =2117.31\;\left[J\right]. \end{array}$$

For dynamic tests, where power will be the comparative criterion, the above graphs will take a slightly different shape, but the advantage of the gantry over the crankset should still be visible.

## 4. Discussion

Analyses carried out on the basis of data from the presented tests have shown that the gantry can receive much more mechanical energy from a human being than a typical crankset. The rotary work for the crankset was 804.81 J for full rotation (360°) of the crankset, while the rotary work for the gantry was equal to 2117.31 J for full rotation (360°) of the first gear wheel. With these data, it is possible to calculate how much better the gantry can receive mechanical energy from a human than a typical crankset. A simple calculation shows that the gantry is 2.63 times better to receive energy from a human than a commonly used crankset.

(14) $$\begin{array}{c} \frac{2117.31\;\left[J\right]}{804.81\;\left[J\right]}≅2.63\;\left[1\right]. \end{array}$$

The chart in Figure 13 shows that while cranking there are large empty areas during which the drive is not transmitted to the bike wheel. What is more, due to the fact that each of the cranks is pressed only by one of the legs, it is not possible to generate such large forces as in the case of the gantry. The hollow areas mainly result from the construction of the crank itself. In horizontal position of cranks, the crankset is not able to generate a torque, even if a force appeared (see positions C1 and C13 in Table 1).

The use of the gantry as a drive allows to generate high forces due to the fact that the bench press is performed with two legs simultaneously. In addition, a large force can be transmitted all the time over the same constant radius, which means that the torque is proportional to the force in the drive system. Some drive downtime will occur as a result of the gantry being retracted but this cannot be determined in static tests and has been omitted.

Despite the fact that several simplifications have been introduced to the analysis, which may slightly distort the results, one can clearly see the large advantage of the gantry used as a drive compared to the crankset.

## 5. Conclusions

Mathematical analysis was performed for one full rotation (360°) of the first gear wheel of the gantry and the crankset. The rotary work for the gantry was 2117.31 J and for the crankset 804.81 J. The gantry can better receive mechanical energy from a human than the crankset. This means that a human will be less tired when riding a horizontal bike equipped with the gantry compared to a horizontal bike equipped with the crankset; assuming that in both cases, the bike speed is the same. Additionally, thanks to the use of the gantry drive in a horizontal bike, it is possible to achieve higher speeds compared to a horizontal bike equipped with the crankset. Dynamic tests are planned in near future.

## Figures and Tables

**Table 1:** Data from analysis and tests for the crankset.

Position number	Angle *γ* [°]	Angle *γ* [rad]	Phase	Co-te: *x*_*c*_ [m]	Co-te: *y*_*c*_ [m]	Angle *α* [°]	Angle *β* [°]	Leg	Measured force *F*_*C*_ [N]	Active radius *r*_*c*_ [m]	Torque *T*_*C*_ [Nm]
C1	0	0	C I	0.00	0.00	53.5	74.2	Left	539.6	0.00	0.0
C2	15	1/12 *π*	C I	0.01	0.04	51.3	76.9	Left	578.8	0.04	25.5
C3	30	1/6 *π*	C I	0.02	0.09	49.1	81.9	Left	667.1	0.09	56.7
C4	45	1/4 *π*	C I	0.05	0.12	51.5	88.7	Left	745.6	0.12	89.6
C5	60	1/3 *π*	C I	0.09	0.15	53.7	95.2	Left	765.2	0.15	112.7
C6	75	5/12 *π*	C I	0.13	0.16	58.7	105.1	Left	873.1	0.16	143.4
C7	90	1/2 *π*	C I	0.17	0.17	61.2	110.5	Left	1265.5	0.17	215.1
C7	90	1/2 *π*	C II	0.17	0.17	61.2	110.5	Left	1265.5	0.17	215.1
C8	105	7/12 *π*	C II	0.21	0.16	66.6	119.4	Left	1559.8	0.16	256.1
C9	120	2/3 *π*	C II	0.26	0.15	73.5	131.8	Left	1706.9	0.15	251.3
C10	135	3/4 *π*	C II	0.29	0.12	80.3	139.6	Left	1746.2	0.12	209.9
C11	150	5/6 *π*	C II	0.32	0.09	87.4	148.2	Left	1667.7	0.09	141.8
C12	165	11/12 *π*	C II	0.33	0.04	93.5	158.9	Left	1569.6	0.04	69.1
C13	180	*π*	C II	0.34	0.00	102.1	170.0	Left	1079.1	0.00	0.0
C13	180	*π*	C III	0.34	0.00	102.1	170.0	Right	1020.2	0.00	0.0
C12	195	13/12 *π*	C III	0.33	0.04	93.5	158.9	Right	1510.7	0.04	66.5
C11	210	7/6 *π*	C III	0.32	0.09	87.4	148.2	Right	1569.6	0.09	133.4
C10	225	5/4 *π*	C III	0.29	0.12	80.3	139.6	Right	1608.8	0.12	193.4
C9	240	4/3 *π*	C III	0.26	0.15	73.5	131.8	Right	1648.1	0.15	242.6
C8	255	17/12 *π*	C III	0.21	0.16	66.6	119.4	Right	1500.9	0.16	246.5
C7	270	3/2 *π*	C III	0.17	0.17	61.2	110.5	Right	1236.1	0.17	210.1
C7	270	3/2 *π*	C IV	0.17	0.17	61.2	110.5	Right	1236.1	0.17	210.1
C6	285	19/12 *π*	C IV	0.13	0.16	58.7	105.1	Right	833.9	0.16	136.9
C5	300	5/3 *π*	C IV	0.09	0.15	53.7	95.2	Right	735.8	0.15	108.3
C4	315	7/4 *π*	C IV	0.05	0.12	51.5	88.7	Right	716.1	0.12	86.1
C3	330	11/6 *π*	C IV	0.02	0.09	49.1	81.9	Right	647.5	0.09	55.0
C2	345	23/12 *π*	C IV	0.01	0.04	51.3	76.9	Right	549.4	0.04	24.2
C1	360	2 *π*	C IV	0.00	0.00	53.5	74.2	Right	519.9	0.00	0.0
	Crank pivot point			0.17	0.00						

**Table 2:** Data from analysis and tests for the gantry.

Position number	Angle *δ* [°]	Angle *δ* [rad]	Phase	Co-te: *x*_*g*_ [m]	Co-te: *y*_*g*_ [m]	Angle *α* [°]	Angle *β* [°]	Leg	Measured force *F*_*G*_ [N]	Active radius *r*_*g*_ [m]	Torque *T*_*G*_ [Nm]
G1	0	0	G I	0.00	0.00	53.5	74.2	Both	1128.2	0.17	191.8
G2	15	1/12 *π*	G I	0.04	0.00	57.5	82.9	Both	1167.4	0.17	198.5
G3	30	1/6 *π*	G I	0.09	0.00	62.4	90.3	Both	1265.5	0.17	215.1
G4	45	1/4 *π*	G I	0.13	0.00	68.3	104.0	Both	1599.0	0.17	271.8
G5	60	1/3 *π*	G I	0.18	0.00	74.1	112.2	Both	2148.4	0.17	365.2
G6	75	5/12 *π*	G I	0.22	0.00	78.7	122.9	Both	2432.9	0.17	413.6
G7	90	1/2 *π*	G I	0.27	0.00	85.1	135.8	Both	3639.5	0.17	618.7
G8	105	7/12 *π*	G I	0.31	0.00	91.5	148.3	Both	2334.8	0.17	396.9
G9	120	2/3 *π*	G I	0.36	0.00	106.0	174.9	Both	1412.6	0.17	240.1
G1	120	2/3 *π*	G II	0.00	0.00	53.5	74.2	Both	1128.2	0.17	191.8
G2	135	3/4 *π*	G II	0.04	0.00	57.5	82.9	Both	1167.4	0.17	198.5
G3	150	5/6 *π*	G II	0.09	0.00	62.4	90.3	Both	1265.5	0.17	215.1
G4	165	11/12 *π*	G II	0.13	0.00	68.3	104.0	Both	1599.0	0.17	271.8
G5	180	*π*	G II	0.22	0.00	74.1	112.2	Both	2148.4	0.17	365.2
G6	195	13/12 *π*	G II	0.27	0.00	78.7	122.9	Both	2432.9	0.17	413.6
G7	210	7/6 *π*	G II	0.31	0.00	85.1	135.8	Both	3639.5	0.17	618.7
G8	225	5/4 *π*	G II	0.36	0.00	91.5	148.3	Both	2334.8	0.17	396.9
G9	240	4/3 *π*	G II	0.00	0.00	106.0	174.9	Both	1412.6	0.17	240.1
G1	240	4/3 *π*	G III	0.00	0.00	53.5	74.2	Both	1128.2	0.17	191.8
G2	255	17/12 *π*	G III	0.04	0.00	57.5	82.9	Both	1167.4	0.17	198.5
G3	270	3/2 *π*	G III	0.09	0.00	62.4	90.3	Both	1265.5	0.17	215.1
G4	285	19/12 *π*	G III	0.13	0.00	68.3	104.0	Both	1599.0	0.17	271.8
G5	300	5/3 *π*	G III	0.18	0.00	74.1	112.2	Both	2148.4	0.17	365.2
G6	315	7/4 *π*	G III	0.22	0.00	78.7	122.9	Both	2432.9	0.17	413.6
G7	330	11/6 *π*	G III	0.27	0.00	85.1	135.8	Both	3639.5	0.17	618.7
G8	345	23/12 *π*	G III	0.31	0.00	91.5	148.3	Both	2334.8	0.17	396.9
G9	360	2 *π*	G III	0.36	0.00	106.0	174.9	Both	1412.6	0.17	240.1

## Data Availability

All relevant data are included in the manuscript.
